# Subchronic inhalation toxicity of gold nanoparticles

**DOI:** 10.1186/1743-8977-8-16

**Published:** 2011-05-14

**Authors:** Jae Hyuck Sung, Jun Ho Ji, Jung Duck Park, Moon Yong Song, Kyung Seuk Song, Hyeon Ryol Ryu, Jin Uk Yoon, Ki Soo Jeon, Jayoung Jeong, Beom Seok Han, Yong Hyun Chung, Hee Kyung Chang, Ji Hyun Lee, Dong Won Kim, Bruce J Kelman, Il Je Yu

**Affiliations:** 1 Toxicity Evaluation Team, Korea Conformity Laboratories, Incheon, Korea; 2 DMC R&D Center, Samsung Electronics Co. Ltd., Suwon, Korea; 3 College of Medicine, Chung-Ang University, Seoul, Korea; 4 R&D Center, HCT Co. Icheon, Korea; 5 National Institute of Food and Drug Safety Research, Korea Food & Drug Administration, Ochang, Korea; 6 Center for Occupational Toxicology, KOSHA, Daejeon, Korea; 7 College of Medicine, Kosin University, Busan, Korea; 8 Veritox, Inc. Seattle, USA; 9 Toxicologcial Research Center, Hoseo University, Asan, Korea

**Keywords:** gold nanoparticles, subchronic inhalation toxicity, tissue distribution, NOAEL

## Abstract

**Background:**

Gold nanoparticles are widely used in consumer products, including cosmetics, food packaging, beverages, toothpaste, automobiles, and lubricants. With this increase in consumer products containing gold nanoparticles, the potential for worker exposure to gold nanoparticles will also increase. Only a few studies have produced data on the *in vivo *toxicology of gold nanoparticles, meaning that the absorption, distribution, metabolism, and excretion (ADME) of gold nanoparticles remain unclear.

**Results:**

The toxicity of gold nanoparticles was studied in Sprague Dawley rats by inhalation. Seven-week-old rats, weighing approximately 200 g (males) and 145 g (females), were divided into 4 groups (10 rats in each group): fresh-air control, low-dose (2.36 × 10^4 ^particle/cm^3^, 0.04 μg/m^3^), middle-dose (2.36 × 10^5 ^particle/cm^3^, 0.38 μg/m^3^), and high-dose (1.85 × 10^6 ^particle/cm^3^, 20.02 μg/m^3^). The animals were exposed to gold nanoparticles (average diameter 4-5 nm) for 6 hours/day, 5 days/week, for 90-days in a whole-body inhalation chamber. In addition to mortality and clinical observations, body weight, food consumption, and lung function were recorded weekly. At the end of the study, the rats were subjected to a full necropsy, blood samples were collected for hematology and clinical chemistry tests, and organ weights were measured. Cellular differential counts and cytotoxicity measurements, such as albumin, lactate dehydrogenase (LDH), and total protein were also monitored in a cellular bronchoalveolar lavage (BAL) fluid. Among lung function test measurements, tidal volume and minute volume showed a tendency to decrease comparing control and dose groups during the 90-days of exposure. Although no statistically significant differences were found in cellular differential counts, histopathologic examination showed minimal alveoli, an inflammatory infiltrate with a mixed cell type, and increased macrophages in the high-dose rats. Tissue distribution of gold nanoparticles showed a dose-dependent accumulation of gold in only lungs and kidneys with a gender-related difference in gold nanoparticles content in kidneys.

**Conclusions:**

Lungs were the only organ in which there were dose-related changes in both male and female rats. Changes observed in lung histopathology and function in high-dose animals indicate that the highest concentration (20 μg/m^3^) is a LOAEL and the middle concentration (0.38 μg/m^3^) is a NOAEL for this study.

## Introduction

Metallic gold (Au°) is arguably considered to be the least corrosive and most biologically inert of all metals [1]. However, that does not mean Au° has been shown to be completely inert in mammals. Human exposure to Au° is common. Much of the earth's population has prolonged dermal contact to metallic gold in the form of jewelry and allergic contact dermatitis (ACD) has been documented as a result of exposure to dental restorations, gold jewelry, and use of gold in food [2-5]. The ingestion of gold-containing liquor beverages has resulted in allergic-type reactions similar to those seen after gold-allergic individuals are exposed to gold through medications or jewelry [3].

The presence of metallic gold has been visualized in human skin biopsies taken from areas of prolonged contact with the metal, such as rings and jewelry, confirming absorption of the solubilized metal even though intact stratum corneum. Skin samples taken from beneath the wedding bands of normal individuals ranged from 0.07 to 0.09 μg of gold per gram of dry weight [6].

Metallic gold can be gradually dissolved by thiol-containing molecules such as cysteine, penicillamine, and glutathione to yield gold (I) complexes [6]. It is possible that the more reactive Au^+ ^and Au^3+ ^species could be a source of the toxicity attributed to Au° [3]. Additionally, metallic gold used in jewelry and prostheses is ordinarily alloyed with other metals; even high-carat yellow gold contains minute quantities of copper and silver, while low-carat gold contains these metals plus zinc and nickel [1].

Gold nanoparticles are widely used in consumer products, including cosmetics, food packaging, beverages, toothpaste, automobiles, and air handling units. Among 1015 consumer nanotechnology-based products, nano-gold-based products rank sixth out of 28 products [7]. In addition, many new nano-gold-based biomedical products are being developed for drug delivery, cancer therapy, diagnostic devices, and biosensing. With this increase in consumer products containing gold nanoparticles, the potential for worker exposure to gold nanoparticles will also increase, which has prompted (in part) a US NTP study of gold nanoparticles [8]. Only a few studies have produced data on the *in vivo *toxicology of gold nanoparticles, meaning that the absorption, distribution, metabolism, and excretion (ADME) of gold nanoparticles remain unclear. This is partially due to the high price of gold in long-term *in vivo *studies. The present subchronic 90-day inhalation toxicity study was designed to identify possible adverse effects and determine a LOAEL or NOAEL. The study was designed according to test guideline 413 from the Organization for Economic Cooperation and Development (OECD) [9], and to comply with Good Laboratory Practices (GLPs). In contrast to silver nanoparticles that are known to be ionized in biological media, gold nanoparticles are not normally ionized in biological media. Thus, inhalation exposure to gold nanoparticles provides the potential to separate the behavior of nanoparticles as particles from chemical effects.

## Materials and methods

### Generation of gold nanoparticles

Gold nanoparticles were generated as described in previous reports [10-13], and the rats exposed in a whole-body-type exposure chamber (1.3 m^3^, Dusturbo, Seoul). The generation system consisted of a small ceramic heater connected to an AC power supply and housed within a quartz tube case. The heater dimensions were 50 × 5 × 1.5 mm^3^, and a surface temperature of about 1500°C within a local heating area of 5 × 10 mm^2 ^was achieved in about 10 seconds [12]. For long-term testing, the source material (about 700 mg of 24 ct gold) was positioned at the highest temperature point. The quartz tube case was 70 mm in diameter and 200 mm long. Clean (dry and filtered) air was used as the carrier gas, and the gas flow maintained at 30.0 L/min (Re = 572, laminar flow regime) using a mass flow controller (MFC, AERA, FC-7810CD-4V, Japan) [10,11]. This generator has already been shown to generate nanoparticles from 1.8 to 6.1 nm (below 10 nm) in diameter that do not agglomerate in air. XRD analysis using an X-ray diffractometer with CuK2 radiation previously showed that the particles generated are metallic gold, not gold oxides [13]. In the current study, the system produced different concentrations of nanoparticles (high, middle, and low) in three separate chambers. For the high-concentration chamber, the nanoparticle generator was operated at 45 L/min (liters per minute) and mixed with 200 L/min of clean ambient air. A portion of the high nanoparticle concentration was then diverted to the middle-concentration chamber using an MFC for the first dilution (1.49 ± 0.06 L/min, mean ± S.E.), and a portion of the middle nanoparticle concentration then diverted to the low-concentration chamber using a second MFC (14.94 ± 0.02 L/min).

### Monitoring of inhalation chamber and analysis of gold nanoparticles

In each chamber, nanoparticle distribution in terms of size was measured directly in real-time using a differential mobility analyzing system (DMAS); combining a differential mobility analyzer (Short type-DMA, 4220, HCT Co., Ltd. Korea, range 2-150 nm) and condensation particle counter (CPC, 4312, HCT Co., Ltd. Korea, 0-10^8^/cm^3^ detection range). The nanoparticles were measured using sheath air at 5 L/min and polydispersed aerosol air at 1 L/min for the DMA and CPC, respectively.

The control chamber was supplied with HEPA-filtered fresh air. Particle concentration in the fresh-air control chamber was measured using a particle sensor (4123, HCT Co., Ltd. Korea) that consisted of two channels; 300-1000 nm and over 1000 nm, to verify the performance of the HEPA filters. Previous studies have shown that the control chamber contains negligible nanoparticles if the HEPA filter is functioning correctly [14,15].

### Transmission Electron Microscopy

The filters used to sample the fume particles were coated with carbon, mounted on an electron microscope grid (200 mesh, Veco, Eerbeek, Holland), and visualized under a field emission-transmission electron microscope (FE-TEM, JEM2100F, JEOL, JAPAN). The diameters of 800 randomly selected particles collected using a nanoparticle collector (nanoparticle collector, 4650, HCT Co., Ltd. Korea) were measured at a magnification of 100,000, and the gold particles analyzed using an energy-dispersive x-ray analyzer (EDX, TM200, Oxford, UK) at an accelerating voltage of 200 kV.

### Animals and Exposure Conditions

Six-week-old male and female, specific-pathogen-free (SPF) Sprague-Dawley rats (Slc:SD) (originally derived from the Charles River SD in 1968) were purchased from SLC (Tokyo, Japan) and acclimated for 1 week before starting the experiments. During the acclimation and experimental periods, the rats were housed in polycarbonate cages (3 rats per cage) in a room with controlled temperature (23 ± 2°C), humidity (55 ± 7%), and a 12-h light/dark cycle. The rats were fed a rodent diet (Harlan Teklab, Plaster International Co., Seoul) and filtered water ad libitum. The 7-week-old rats, weighed approximately 200 g for the males and 145 g for the females at the beginning of exposure. The rats were divided into 4 groups (10 rats in each group/sex): fresh-air control, low-dose group (target dose, 2.5 × 10^4^ particles/cm^3^, 1.0 × 10^6^ nm^2^/cm^3^ surface area), middle-dose group (target dose, 2.5 × 10^5^ particles/cm^3^, 6.0 × 10^6^ nm^2^/cm^3^ surface area), and high-dose group (target dose, 1.2-2.8 × 10^6^ particles/cm^3^, 3.0 × 10^8^ nm^2^/cm^3^ surface area). The animals were exposed to the gold nanoparticles for 6 hours/day, 5 days/week, for 13-weeks, and housed in individual wire cages without food and water during the 6-hour exposure periods. The animals were examined daily on weekdays for any evidence of exposure-related effects, including respiratory, dermal, behavioral, nasal, or genitourinary changes suggestive of irritation. Body weights were evaluated at the time of purchase, at the time of grouping, after one day of exposure, once a week during exposure, and before necropsy. The experiment was approved by the KCL Institutional Animal Care and Use Committee.

### Biochemistry and Hematology

At the conclusion of the 90-day experiment, the rats were 20 wks old. Before necropsy, food was withheld for 24 h and the rats were anesthetized with an overdose of sodium pentobarbital. Blood was then drawn from the abdominal aorta, collected in vacutainers, and analyzed for ALB (albumin), ALP (alkaline phosphatase), Ca (calcium), CHO (cholesterol), CRE (creatinine), gamma-GT (gamma-glutamyl transpeptidase), GLU (glucose), GOT (glutamic oxaloacetic transaminase), GPT (glutamic pyruvic transaminase), IP (inorganic phosphorus), LDH (lactate dehydrogenase), MG (magnesium), TP (total protein), UA (uric acid), BUN (blood urea nitrogen), TBIL (total bilirubin), CK (creatine phosphokinase), Na (sodium), K (potassium), Cl (chloride), TG (triglyceride), and A/G (ratio of albumin to globulin) using a biochemical blood analyzer (Hitachi 7180, Hitachi, Japan). The blood was also analyzed for the WBC (white blood cell count), RBC (red blood cell count), Hb (hemoglobin concentration), HTC (hematocrit), MCV (mean corpuscular volume), MCH (mean corpuscular hemoglobin), MCHC (mean corpuscular hemoglobin concentration), RDW (red cell distribution width), PLT (platelet count), MPV (mean platelet volume), NE# (number of neutrophils), NE% (percent of neutrophils), LY# (number of lymphocytes), LY% percent of lymphocytes), MO# (number of monocytes), MO% (percent of monocytes), EO# (number of eosinophils), EO% (percent of eosinophils), BA# (number of basophils), and BA% (percent of basophils) using a blood cell counter (Hemavet 0950, CDC Tech., USA).

### Erythrocyte aggregation test

To evaluate aggregation of red blood cells or blood coagulation attributable to the gold nanoparticles, 3.2% sodium citrate was used for anticoagulation, and the activated partial thromboplastin time (APPT) and prothrombin time (PT) measured using a blood coagulation analyzer (ACL 7000, Instrumentation Laborato Co., U.S.A.).

### Kidney function test

Since a gender difference in silver accumulation was previously noted in kidneys [14,15], kidney function was measured based on the N-acetyl-beta-D-glucosaminidase (NAG) and protein in the urine using metabolic cages for 5 rats from each exposure group and the control.

### Organ Weights and Histopathology

After collecting blood samples, rats were killed by cervical dislocation, and all the organs carefully removed, including the adrenal glands, bladder, testes, ovaries, uterus, epididymis, seminal vesicle, heart, thymus, thyroid gland, trachea, esophagus, tongue, prostate, lungs, nasal cavity, kidneys, spleen, liver, pancreas, brain and olfactory bulb. The organs were then weighed, fixed in a 10% formalin solution containing neutral phosphate-buffered saline, embedded in paraffin, stained with hematoxylin and eosin, and examined under light microscopy.

### Lung Function Testing

The lung function of four rats from each dose group was evaluated every week during the 90-day exposure using a ventilated bias flow whole-body plethysmograph (WBP) (SFT3816, Buxco Electronics, Sharon, CT) that consisted of a reference chamber and animal chamber interconnected by a pressure transducer (MAX1320, Buxco Electronics, Sharon, CT; [15]). Measurements taken during pulmonary function testing included the tidal volume (TV, ml), minute volume (MV, ml/min), respiratory frequency (BPM, breath/min), inspiration time (Ti, s), expiration time (Te, s), peak inspiration flow (PIF, ml/s), and peak expiration flow (PEF, ml/s). After the last 6-hour exposure, the rats were placed in an animal chamber, left for 40 min to stabilize, and measurements were taken in the whole-body plethysmograph for 5 minutes.

### Bronchoalveolar Lavage (BAL) Cell Evaluation

The same animals that were used for lung function testing were also subjected to a BAL at the end of the 90-day exposure period. The rats were deeply anesthetized with an overdose of sodium pentobarbital. Blood was collected from the abdominal aorta and the rats were then exsanguinated by severing the abdominal aorta. The lungs were lavaged 14 times with 3 ml aliquots of a warm calcium- and magnesium-free phosphate buffer solution (PBS), pH 7.4. The samples were also centrifuged for 7 min at 500 × g and the cell-free BAL fluid discarded. The cell pellets from all washes for each rat were then combined, washed, and resuspended in 1 ml of a phosphate buffered saline (PBS) buffer and evaluated [16-20]. Total cell numbers were determined using a hemacytometer. The cells were first smeared and then stained with the Wright Giemsa Sure Stain [21] to allow a count of the total number of cells, macrophages, polymorphonuclear cells (PMNs), and lymphocytes. BAL levels of total protein, albumin, and LDH were also measured using a blood biochemical analyzer (Hitachi 7180, Hitachi, Japan).

### Determination of Tissue Gold

After necropsy, samples of the blood (0.5 ml), liver, lungs, brain, kidneys, and olfactory bulb were analyzed to determine organ distribution of gold. Care was taken to avoid contaminating tissues with gold from the fur and skin of each animal. After wet digestion using a flameless method, the tissue concentrations of gold were analyzed using an atomic absorption spectrophotometer equipped with a Zeeman graphite furnace (Perkin Elmer 5100ZL, Zeeman furnace module, USA) based on the NIOSH 7300 method [22]. The detection limit was 2 ppb and the limit of quantification was 7 ppb.

### Statistical analysis

All results are expressed as means ± standard error (SE). An analysis of variance (ANOVA) test and Duncan's multiple range tests were used to compare the body weights, bronchoalveolar lavage cell distributions, lung function test parameters, and all other comparisons for the three dose groups with those for the control rats. Histopatholgic results were analyzed using a Chi-square analysis. The level of significance was set at p < 0.05 and p < 0.01.

## Results

### Gold nanoparticle distribution

**Figure 1:**
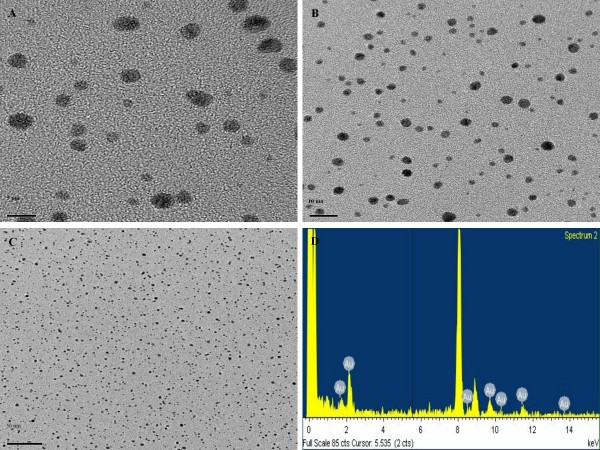
**TEM morphology of gold nanoparticles and EDX spectrometer pattern**. (A-C) Field Emission-Transmission Electron Microscope (× 100,000), (D) EDX spectrometer

**Figure 2:**
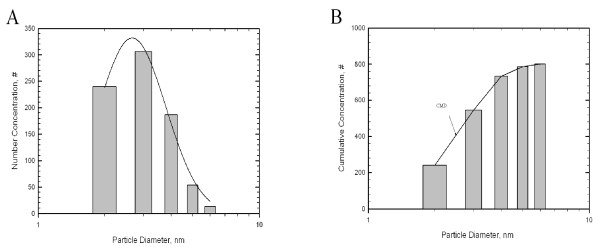
**Distribution of gold nanoparticles**. Cumulative median diameter (CMD). A, number concentration; B, cumulative concentration

**Figure 3:**
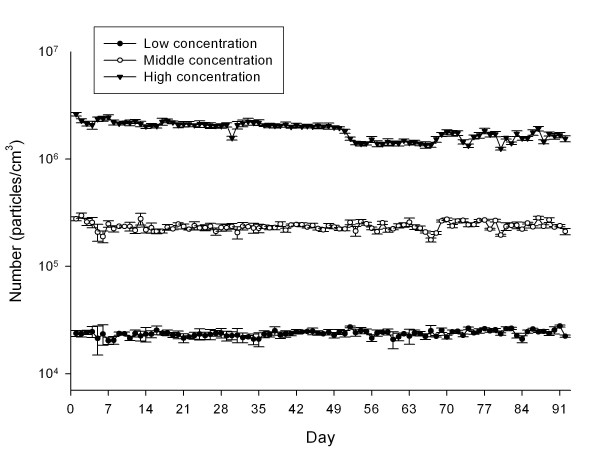
**Distribution of gold nanoparticle concentrations during 13-week exposure period**.

Nanoparticle distributions in chambers are shown in Table 1. For the high-concentration chamber, the geometric mean diameter (GMD), concentration, and surface area of the gold nanoparticles measured by the DMAS were 5.06 nm, 1.85 × 10^6 ^particles/cm^3^, 20.02 μg/m^3^, and 3.64 × 10^8 ^nm^2^/cm^3^, respectively; for the middle-concentration chamber were 4.12 nm, 2.36 × 10^5 ^particles/cm^3^, 0.38 μg/m^3 ^and 1.68 × 10^7 ^nm^2^/cm^3^, respectively, and for the low-concentration chamber were 4.3 nm, 2.36 × 10^4 ^particles/cm^3^, 0.04 μg/m^3^, and 1.9 × 10^6 ^nm^2^/cm^3^, respectively. The gold nanoparticles observed by TEM were spheroid in shape in both non-aggregated and non-agglomerated forms, with diameters under 6 nm (Figure 1,A-C). TEM-EDX analysis indicated that only elemental gold was present (Figure 1D). The diameters were log normally distributed between 1 and 6 nm, and the CMD (count median diameter) and GSD were 2.47 nm and 1.42, respectively (Figure 2). The distribution of gold nanoparticles was well maintained during the 90-day exposure period, as shown in Figure 3.

**Table 1:** Distribution of gold nanoparticles (mean ± S.E.).

Group	Site	Diameter^†^(nm)	Number (particles/cm^3^)	Surface (nm^2^/cm^3^)	Mass (μg/m^3^)
Control		0	0	0	0

	Up^a^	4.29(1.69)	2.46 × 10^4 ^± 2.38 × 10^2^	1.96 × 10^6 ^± 3.28 × 10^4^	0.04 ± 0.00
	
Low	Down^b^	4.32(1.64)	2.21 × 10^4 ^± 2.98 × 10^2^	1.81 × 10^6 ^± 4.03 × 10^4^	0.04 ± 0.00
	
	Total	4.30(1.67)	2.36 × 10^4 ^± 1.90 × 10^2^	1.90 × 10^6 ^± 2.55 × 10^4^	0.04 ± 0.00

	Up	4.11(1.35)	2.40 × 10^5 ^± 1.98 × 10^3^	1.71 × 10^7 ^± 2.24 × 10^5^	0.38 ± 0.02
	
Middle	Down	4.13(1.41)	2.29 × 10^5 ^± 2.59 × 10^3^	1.53 × 10^7 ^± 2.05 × 10^5^	0.38 ± 0.03
	
	Total	4.12(1.38)	2.36 × 10^5 ^± 2.32 × 10^3^	1.68 × 10^7 ^± 1.66 × 10^5^	0.38 ± 0.02

	Up	4.96(1.77)	1.98 × 10^6 ^± 1.85 × 10^4^	3.67 × 10^8 ^± 6.35 × 10^7^	20.14 ± 0.64
	
High	Down	5.20(1.93)	1.66 × 10^6 ^± 1.53 × 10^4^	3.60 × 10^8 ^± 7.51 × 10^6^	20.80 ± 0.73
	
	Total	5.06(1.86)	1.85 × 10^6 ^± 1.36 × 10^4^	3.64 × 10^8 ^± 4.85 × 10^6^	20.02 ± 0.41

†: GM(GSD)

a: breathing zone,

b: below breathing zone.

Control chamber indicates number of particles passed after HEPA filter

### Animal Observation, Food Consumption, and Effect on Body and Organ Weights

**Figure 4:**
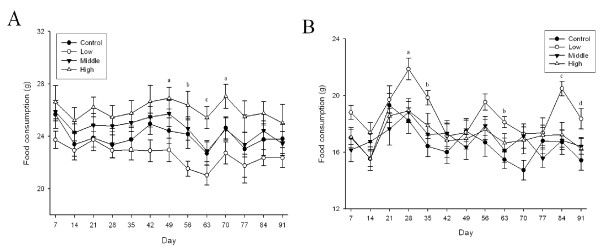
**Food intake change during 90-day exposure period**. A, male; B, female. (A. a, p < 0.05 high group vs. low group; b, p < 0.01 middle and high groups vs. low group; c, p < 0.01 high group vs. other groups. B. a, p < 0.05 low group vs. other groups; b. p < 0.05 low group vs. control and middle groups; c, p < 0.01 low group vs. other groups; d, p < 0.05 low group vs. control and high groups)

**Figure 5:**
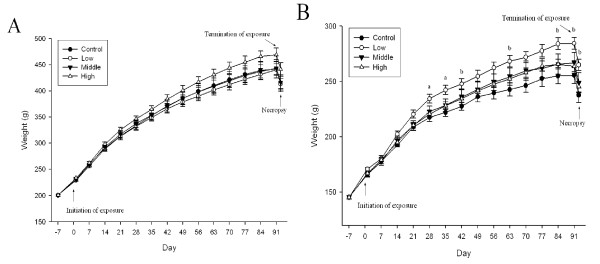
**Body weight changes for rats exposed to gold nanoparticles**. A, male; B, female (a, p < 0.05 low group vs. other groups; b, p < 0.05 low group vs. control group)

No significant gross effects were observed during the 90-day exposure period. There was a loss of hair on the front left leg of one male in the high dose group at 9 weeks and a loss of hair on both front legs of one female in the high dose group at 12 weeks of exposure. There was no apparent reason for the loss of hair in these two animals. In our experience, hair loss in isolated animals is not an unusual event. Examination of the eyes of control and high-dose animals did not reveal any effects. Food intake increased in the high-dose male group when compared to the control (p < 0.05), middle-dose (p < 0.05), and low-dose (p < 0.01) groups (Figure 4A). Food intake also increased in the low-dose female group when compared to the control (p < 0.01), middle-dose (p < 0.01), and high-dose (p < 0.05) groups (Figure 4B). While there were no significant changes in the body weights of the male rats (Figure 5A), there was a significant body weight gain in the low-dose female group (p < 0.05) when compared to the control, middle-dose, and high-dose groups after 4 weeks of exposure (Figure 5B). There was a 0.3 × 0.2 cm white mass in the right kidney of one rat from the high-dose group. No significant organ weight changes were observed in either the male or female rats at the conclusion of the study (Table 2 and 3).

**Table 2:** Relative organ weights of male rats (% body weight)

GROUP: (mean ± S.E)	Control	Low	Middle	High
Body weight	415.08 ± 7.79 (10)	412.81 ± 10.83 (10)	415.93 ± 11.72 (10)	440.81 ± 12.48 (10)
Testis (Left)	0.41 ± 0.01 (8)	0.44 ± 0.02 (10)	0.45 ± 0.01 (10)	0.41 ± 0.02 (8)
Testis (Right)	0.40 ± 0.01 (8)	0.43 ± 0.02 (10)	0.45 ± 0.01 (10)	0.40 ± 0.02 (8)
Kidney (Left)	0.27 ± 0.01 (8)	0.28 ± 0.00 (10)	0.29 ± 0.01 (10)	0.26 ± 0.01 (8)
Kidney (Right)	0.28 ± 0.01 (8)	0.29 ± 0.01 (10)	0.29 ± 0.01 (10)	0.28 ± 0.01 (8)
Spleen	0.17 ± 0.01 (8)	0.17 ± 0.01 (10)	0.17 ± 0.01 (10)	0.17 ± 0.01 (8)
Liver	2.60 ± 0.05 (8)	2.52 ± 0.05 (10)	2.61 ± 0.05 (10)	2.56 ± 0.05 (8)
Adrenal gland (Left)	0.007 ± 0.000 (8)	0.007 ± 0.001 (10)	0.007 ± 0.000 (10)	0.007 ± 0.000 (8)
Adrenal gland (Right)	0.007 ± 0.000 (8)	0.007 ± 0.000 (10)	0.007 ± 0.001 (10)	0.007 ± 0.001 (8)
Heart	0.27 ± 0.01 (8)	0.28 ± 0.00 (10)	0.28 ± 0.01 (10)	0.27 ± 0.00 (8)
Thymus	0.08 ± 0.01 (8)	0.08 ± 0.00 (10)	0.09 ± 0.01 (10)	0.08 ± 0.01 (8)
Lung (Left)	0.12 ± 0.00 (6)	0.12 ± 0.00 (6)	0.11 ± 0.00 (6)	0.11 ± 0.00 (6)
Kung (Right)	0.22 ± 0.00 (6)	0.23 ± 0.00 (6)	0.21 ± 0.00 (6)	0.21 ± 0.00 (6)
Brain	0.49 ± 0.01 (8)	0.51 ± 0.01 (10)	0.51 ± 0.01 (10)	0.47 ± 0.01 (8)
Olfactory bulb	0.017 ± 0.002 (8)	0.027 ± 0.002 (10)	0.023 ± 0.003 (10)	0.022 ± 0.001 (8)
Hypophysis	0.002 ± 0.000 (8)	0.002 ± 0.000 (10)	0.002 ± 0.000 (10)	0.002 ± 0.000 (8)

( ): number of animals

**Table 3:** Relative organ weights of female rats (% body weight)

GROUP: (mean ± S.E)	Control	Low	Middle	High
Body weight	237.53 ± 6.76 (10)	264.88 ± 5.19 (10)	248.82 ± 9.05 (10)	245.26 ± 4.91 (10)
Ovary (Left)	0.020 ± 0.002 (10)	0.021 ± 0.001 (10)	0.019 ± 0.001 (10)	0.020 ± 0.001 (10)
Ovary (Right)	0.021 ± 0.001 (10)	0.021 ± 0.001 (10)	0.021 ± 0.001 (10)	0.021 ± 0.002 (10)
Kidney (Left)	0.29 ± 0.01 (10)	0.28 ± 0.00 (10)	0.30 ± 0.01 (10)	0.29 ± 0.01 (10)
Kidney (Right)	0.30 ± 0.01 (10)	0.29 ± 0.01 (10)	0.30 ± 0.01 (10)	0.29 ± 0.01 (10)
Spleen	0.20 ± 0.01 (10)	0.18 ± 0.01 (10)	0.19 ± 0.01 (10)	0.18 ± 0.01 (10)
Liver	2.42 ± 0.03 (10)	2.43 ± 0.04 (10)	2.54 ± 0.06 (10)	2.44 ± 0.05 (10)
Adrenal gland (Left)	0.013 ± 0.001 (10)	0.013 ± 0.001 (10)	0.012 ± 0.001 (10)	0.013 ± 0.001 (10)
Adrenal gland (Right)	0.013 ± 0.001 (10)	0.013 ± 0.001 (10)	0.012 ± 0.001 (10)	0.015 ± 0.002 (10)
Heart	0.34 ± 0.01 (10)	0.32 ± 0.01 (10)	0.32 ± 0.01 (10)	0.33 ± 0.01 (10)
Thymus	0.12 ± 0.00 (10)	0.14 ± 0.01 (10)	0.12 ± 0.01 (10)	0.13 ± 0.01 (10)
Lung (Left)	0.18 ± 0.03 (6)	0.17 ± 0.03 (6)	0.17 ± 0.02 (6)	0.16 ± 0.00 (6)
Kung (Right)	0.36 ± 0.05 (6)	0.31 ± 0.03 (6)	0.32 ± 0.04 (6)	0.30 ± 0.01 (6)
Brain	0.84 ± 0.02 (10)	0.76 ± 0.02 (10)	0.80 ± 0.03 (10)	0.82 ± 0.02 (10)
Olfactory bulb	0.040 ± 0.003 (10)	0.034 ± 0.002 (10)	0.041 ± 0.002 (10)	0.039 ± 0.002 (10)
Hypophysis	0.005 ± 0.001 (10)	0.005 ± 0.000 (10)	0.006 ± 0.001 (10)	0.006 ± 0.001 (10)

( ): number of animals

### Effects on Hematology and Blood Biochemistry

No significant dose-related differences were observed for the hematology values (Table 4 and 5) and blood biochemical measurements (Table 6 and 7).

**Table 4:** Hematological values for male rats

GROUP: (mean ± S.E)	Control	Low	Middle	High
WBC^1 ^(K/μL)	5.17 ± 0.44 (8)	6.42 ± 0.64 (10)	4.80 ± 0.27 (10)	4.67 ± 0.20 (8)
NE^2 ^(K/μL)	1.56 ± 0.14 (8)	1.67 ± 0.20 (10)	1.41 ± 0.11 (10)	1.41 ± 0.10 (8)
LY^3 ^(K/μL)	3.36 ± 0.36 (8)	4.41 ± 0.42 (10)	3.11 ± 0.22 (10)	3.06 ± 0.18 (8)
MO^4 ^(K/μL)	0.24 ± 0.03 (8)	0.32 ± 0.04 (10)	0.27 ± 0.17 (10)	0.19 ± 0.02 (8)
EO^5 ^(K/μL)	0.005 ± 0.002 (8)	0.018 ± 0.013 (10)	0.006 ± 0.002 (10)	0.014 ± 0.003 (8)
BA^6 ^(K/μL)	0.000 ± 0.000 (8)	0.008 ± 0.008 (10)	0.000 ± 0.000 (10)	0.003 ± 0.002 (8)
NE^7 ^(%)	30.72 ± 2.39 (8)	25.71 ± 0.98 (10)	29.553 ± 1.70 (10)	30.28 ± 2.07 (8)
LY^8 ^(%)	64.45 ± 2.38 (8)	68.97 ± 1.18 (10)	64.52 ± 1.77 (10)	65.39 ± 1.96 (8)
MO^9 ^(%)	4.71 ± 0.50 (8)	5.05 ± 0.48 (10)	5.78 ± 0.42 (10)	4.00 ± 0.27 (8)
EO^10 ^(%)	0.10 ± 0.03 (8)	0.20 ± 0.11 (10)	0.15 ± 0.05 (10)	0.30 ± 0.05 (8)
BA^11 ^(%)	0.15 ± 0.01 (8)	0.08 ± 0.07 (10)	0.01 ± 0.00 (10)	0.03 ± 0.02 (8)
RBC^12 ^(M/μL)	8.87 ± 0.11 (8)	8.67 ± 0.33 (10)	9.18 ± 0.16 (10)	9.09 ± 0.17 (8)
Hb^13 ^(g/dL)	17.68 ± 0.23 (8)	17.33 ± 0.18 (10)	18.26 ± 0.24^c ^(10)	17.91 ± 0.22 (8)
HCT^14 ^(%)	36.71 ± 1.07 (8)	36.68 ± 0.95 (10)	38.26 ± 0.98 (10)	37.66 ± 1.18 (8)
MCV^15 ^(fL)	41.35 ± 0.97 (8)	42.30 ± 0.86 (10)	41.65 ± 0.78 (10)	41.46 ± 1.13 (8)
MCH^16 ^(pg)	19.95 ± 0.34 (8)	20.00 ± 0.25 (10)	19.92 ± 0.36 (10)	19.73 ± 0.28 (8)
MCHC^17 ^(g/dL)	48.39 ± 1.29 (8)	47.53 ± 1.31 (10)	47.98 ± 1.32 (10)	47.86 ± 1.44 (8)
RDW^18 ^(%)	20.74 ± 0.94 (8)	21.24 ± 1.08 (10)	21.76 ± 1.09 (10)	20.84 ± 0.97 (8)
PLT^19 ^(K/μL)	949.88 ± 50.68 (8)	862.70 ± 41.33 (10)	894.30 ± 32.72 (10)	882.38 ± 42.43 (8)
MPV^20 ^(fL)	6.43 ± 0.09 (8)	6.67 ± 0.12 (10)	6.61 ± 0.11 (10)	6.44 ± 0.16 (8)

( ): number of animals

1, White blood cells; 2, Neutrophils; 3, Lymphocytes; 4, Monocytes; 5, Eosinophils; 6, Basophils; 7, Percent of neutrophils; 8, Percent of lymphocytes; 9, Percent of monocytes; 10, Percent of eosinophils; 11, Percent of basophils; 12, Red blood cells; 13, Hemoglobin; 14, Hematocrits; 15, Mean corpuscular volume; 16, Mean corpuscular hemoglobin; 17, Mean corpuscular hemoglobin concentration; 18, Red cell distribution width; 19, Platelet; 20, Mean platelet volume

**Table 5:** Hematological values for female rats

GROUP: (mean ± S.E)	Control	Low	Middle	High
WBC^1 ^(K/μL)	3.60 ± 0.43 (10)	2.99 ± 0.30 (10)	3.25 ± 0.36 (10)	3.39 ± 0.39 (10)
NE^2 ^(K/μL)	0.96 ± 0.11 (10)	0.81 ± 0.08 (10)	0.79 ± 0.08 (10)	1.02 ± 0.15 (10)
LY^3 ^(K/μL)	2.50 ± 0.33 (10)	2.06 ± 0.22 (10)	2.31 ± 0.28 (10)	2.24 ± 0.25 (10)
MO^4 ^(K/μL)	0.13 ± 0.03 (10)	0.12 ± 0.02 (10)	0.14 ± 0.02 (10)	0.12 ± 0.02 (10)
EO^5 ^(K/μL)	0.007 ± 0.004 (10)	0.004 ± 0.002 (10)	0.006 ± 0.002 (10)	0.006 ± 0.002 (10)
BA^6 ^(K/μL)	0.002 ± 0.002 (10)	0.000 ± 0.000 (10)	0.001 ± 0.001 (10)	0.000 ± 0.000 (10)
NE^7 ^(%)	27.22 ± 2.34 (10)	27.42 ± 1.37 (10)	24.97 ± 1.43 (10)	29.81 ± 1.87 (10)
LY^8 ^(%)	26.99 ± 2.13 (10)	68.23 ± 1.57 (10)	70.62 ± 1.40 (10)	66.50 ± 1.68 (10)
MO^9 ^(%)	3.47 ± 0.43 (10)	4.16 ± 0.51 (10)	4.17 ± 0.31 (10)	3.49 ± 0.53 (10)
EO^10 ^(%)	0.24 ± 0.09 (10)	0.17 ± 0.04 (10)	0.20 ± 0.05 (10)	0.18 ± 0.04 (10)
BA^11 ^(%)	0.08 ± 0.04 (10)	0.02 ± 0.01 (10)	0.04 ± 0.02 (10)	0.03 ± 0.01 (10)
RBC^12 ^(M/μL)	8.19 ± 0.09 (10)	7.94 ± 0.15 (10)	8.07 ± 0.05 (10)	7.96 ± 0.11 (10)
Hb^13 ^(g/dL)	16.99 ± 0.22 (10)	16.70 ± 0.28 (10)	16.74 ± 0.12 (10)	16.61 ± 0.27 (10)
HCT^14 ^(%)	39.93 ± 0.67 (10)	39.41 ± 0.71 (10)	39.54 ± 0.41 (10)	39.24 ± 0.71 (10)
MCV^15 ^(fL)	48.82 ± 0.86 (10)	49.70 ± 0.39 (10)	48.99 ± 0.65 (10)	49.30 ± 0.50 (10)
MCH^16 ^(pg)	20.77 ± 0.29 (10)	21.06 ± 0.22 (10)	20.74 ± 0.18 (10)	20.87 ± 0.19 (10)
MCHC^17 ^(g/dL)	42.58 ± 0.37 (10)	42.38 ± 0.31 (10)	42.37 ± 0.36 (10)	42.35 ± 0.26 (10)
RDW^18 ^(%)	22.75 ± 1.22 (10)	22.58 ± 1.27 (10)	22.52 ± 1.10 (10)	23.94 ± 1.34 (10)
PLT^19 ^(K/μL)	693.10 ± 68.03 (10)	762.70 ± 25.24 (10)	729.00 ± 15.91 (10)	764.90 ± 14.86 (10)
MPV^20 ^(fL)	6.45 ± 0.15 (10)	6.66 ± 0.17 (10)	6.47 ± 0.14 (10)	6.36 ± 0.20 (10)

( ): number of animals

1, White blood cells; 2, Neutrophils; 3, Lymphocytes; 4, Monocytes; 5, Eosinophils; 6, Basophils; 7, Percent of neutrophils; 8, Percent of lymphocytes; 9, Percent of monocytes; 10, Percent of eosinophils; 11, Percent of basophils; 12, Red blood cells; 13, Hemoglobin; 14, Hematocrits; 15, Mean corpuscular volume; 16, Mean corpuscular hemoglobin; 17, Mean corpuscular hemoglobin concentration; 18, Red cell distribution width; 19, Platelet; 20, Mean platelet volume

**Table 6:** Serum biochemical values for male rats

GROUP: (mean ± S.E)	Control	Low	Middle	High
ALB^1 ^(g/dL)	2.43 ± 0.02 (8)	2.42 ± 0.02 (10)	2.50 ± 0.03 (10)	2.56 ± 0.05 (8)
ALP^2 ^(IU/L)	286.63 ± 13.86 (8)	299.10 ± 17.32 (10)	332.30 ± 17.13 (10)	292.88 ± 121.40 (8)
CA^3 ^(mg/dL)	9.18 ± 0.12 (8)	9.09 ± 0.04 (10)	15.02 ± 0.75b (10)	18.93 ± 0.14 (8)
CHO^4 ^(mg/dL)	68.75 ± 4.69 (8)	69.60 ± 4.76 (10)	69.00 ± 3.44 (10)	77.38 ± 8.66 (8)
CRE^5 ^(mg/dL)	0.53 ± 0.05 (8)	0.59 ± 0.13 (10)	1.08 ± 0.08b (10)	0.91 ± 0.12 (8)
γ-GT^6 ^(IU/L)	1.38 ± 0.18 (8)	1.10 ± 0.18 (10)	1.10 ± 0.18 (10)	1.25 ± 0.16 (8)
GLU^7 ^(mg/dL)	158.13 ± 9.34 (8)	175.40 ± 9.56 (10)	188.50 ± 13.55 (10)	162.38 ± 7.81 (8)
GOT^8 ^(IU/L)	124.25 ± 16.27 (8)	105.10 ± 8.97 (10)	108.60 ± 9.89 (10)	102.13 ± 14.7 (8)
GPT^9 ^(IU/L)	66.63 ± 14.49 (8)	49.30 ± 2.50 (10)	54.90 ± 4.18 (10)	51.50 ± 6.76 (8)
IP^10 ^(mg/dL)	6.54 ± 0.31 (8)	6.73 ± 0.24 (10)	6.60 ± 0.20 (10)	6.20 ± 0.23 (8)
LDH^11 ^(IU/L)	562.63 ± 103.39 (8)	555.50 ± 149.47 (10)	592.40 ± 179.20 (10)	538.88 ± 193.15 (8)
MG^12 ^(mg/dL)	2.04 ± 0.04 (8)	2.12 ± 0.05 (10)	2.05 ± 0.05 (10)	2.03 ± 0.05 (8)
TP^13 ^(g/dL)	6.00 ± 0.07 (8)	5.92 ± 0.02 (10)	6.11 ± 0.05 (10)	6.25 ± 0.09 (8)
UA^14 ^(mg/dL)	1.24 ± 0.21 (8)	1.39 ± 0.10 (10)	1.62 ± 0.26 (10)	1.04 ± 0.10 (8)
BUN^15 ^(mg/dL)	15.88 ± 0.65 (8)	16.86 ± 0.47 (10)	16.33 ± 0.48 (10)	15.11 ± 0.37 (8)
TBIL^16 ^(mg/dL)	0.013 ± 0.004 (8)	0.008 ± 0.005 (10)	0.001 ± 0.008 (10)	0.010 ± 0.008 (8)
TG^17 ^(mg/dL)	37.25 ± 6.87 (8)	40.60 ± 9.24 (10)	38.80 ± 6.00 (10)	41.75 ± 5.54 (8)
CK^18 ^(IU/L)	543.88 ± 77.39 (8)	1001.20 ± 213.75 (10)	759.70 ± 146.20 (10)	419.13 ± 125.29 (8)
Na^19 ^(mmol/L)	129.13 ± 0.35 (8)	129.30 ± 0.52 (10)	128.50 ± 0.56 (10)	132.13 ± 2.07 (8)
K^20 ^(mmol/L)	2.80 ± 0.07 (8)	3.02 ± 0.10 (10)	3.21 ± 0.15 (10)	2.80 ± 0.10 (8)
Cl^21 ^(mmol/L)	97.25 ± 0.31 (8)	97.70 ± 0.21 (10)	97.10 ± 0.35 (10)	99.75 ± 1.49 (8)

( ): number of animals

1, Albumin; 2, Alkaline phosphatase; 3, Calcium; 4, Cholesterol; 5, Creatinine; 6, Gamma glutamyl transpeptidase; 7, Glucose; 8, Glutamic oxalacetic transaminase; 9, Glutamic pyruvic transaminase; 10, Inorganic phosphorus; 11, Lactate Dehydrogenase; 12, Magnesium; 13, Total protein; 14, Uric acid; 15, Blood urea nitrogen; 16, Total bilirubin; 17, Triglyceride; 18, Creatine Kinase; 19, Sodium; 20, Potassium; 21, Chloride

**Table 7:** Serum biochemical values for female rats

GROUP: (mean ± S.E)	Control	Low	Middle	High
ALB^1 ^(g/dL)	2.77 ± 0.07 (10)	2.76 ± 0.08 (10)	3.18 ± 0.11 (10)	2.85 ± 0.06 (10)
ALP^2 ^(IU/L)	223.20 ± 16.90 (10)	200.80 ± 22.94 (10)	167.70 ± 9.59 (10)	227.20 ± 13.71 (10)
CA^3 ^(mg/dL)	10.02 ± 0.10 (10)	10.10 ± 0.14 (10)	10.36 ± 0.17 (10)	10.29 ± 0.19 (10)
CHO^4 ^(mg/dL)	89.60 ± 4.49 (10)	96.90 ± 4.66 (10)	105.20 ± 5.59 (10)	92.70 ± 5.18 (10)
CRE^5 ^(mg/dL)	0.78 ± 0.04 (10)	0.89 ± 0.04 (10)	0.89 ± 0.07 (10)	0.85 ± 0.03 (10)
γ-GT^6 ^(IU/L)	0.10 ± 0.10 (10)	0.20 ± 0.13 (10)	0.00 ± 0.00 (10)	0.40 ± 0.22 (10)
GLU^7 ^(mg/dL)	134.70 ± 5.24 (10)	143.30 ± 4.82 (10)	154.50 ± 8.04 (10)	135.90 ± 5.23 (10)
GOT^8 ^(IU/L)	69.00 ± 2.21 (10)	141.20 ± 48.60 (10)	77.40 ± 4.96 (10)	82.30 ± 5.17 (10)
GPT^9 ^(IU/L)	33.80 ± 1.13 (10)	64.20 ± 15.61 (10)	40.60 ± 2.85 (10)	41.60 ± 2.63 (10)
IP^10 ^(mg/dL)	5.66 ± 0.23 (10)	5.64 ± 0.49 (10)	5.58 ± 0.40 (10)	5.55 ± 0.29 (10)
LDH^11 ^(IU/L)	180.40 ± 27.93 (10)	254.90 ± 49.86 (10)	222.70 ± 22.46 (10)	216.40 ± 30.50 (10)
MG^12 ^(mg/dL)	2.06 ± 0.04 (10)	2.04 ± 0.04 (10)	2.13 ± 0.07 (10)	2.03 ± 0.04 (10)
TP^13 ^(g/dL)	6.20 ± 0.09 (10)	6.07 ± 0.17 (10)	6.76 ± 0.18 (10)	6.40 ± 0.13 (10)
UA^14 ^(mg/dL)	1.07 ± 0.11 (10)	1.22 ± 0.09 (10)	1.14 ± 0.06 (10)	0.99 ± 0.08 (10)
BUN^15 ^(mg/dL)	17.86 ± 0.74 (10)	18.08 ± 0.61 (10)	18.99 ± 0.90 (10)	17.52 ± 0.87 (10)
TBIL^16 ^(mg/dL)	0.005 ± 0.015 (10)	0.009 ± 0.013 (10)	0.020 ± 0.005 (10)	0.024 ± 0.005 (10)
TG^17 ^(mg/dL)	8.50 ± 1.28 (10)	9.40 ± 0.93 (10)	11.20 ± 1.83 (10)	6.90 ± 0.57 (10)
CK^18 ^(IU/L)	269.20 ± 64.71 (10)	265.50 ± 24.99 (10)	341.30 ± 68.11 (10)	212.30 ± 26.38 (10)
Na^19 ^(mmol/L)	136.50 ± 0.48 (10)	139.20 ± 2.80 (10)	138.90 ± 0.95 (10)	138.50 ± 1.26 (10)
K^20 ^(mmol/L)	3.69 ± 0.12 (10)	3.56 ± 0.10 (10)	3.62 ± 0.07 (10)	3.48 ± 0.09 (10)
Cl^21 ^(mmol/L)	102.00 ± 0.33 (10)	103.40 ± 2.05 (10)	103.00 ± 0.86 (10)	102.80 ± 1.17 (10)

( ): number of animals

1, Albumin; 2, Alkaline phosphatase; 3, Calcium; 4, Cholesterol; 5, Creatinine; 6, Gamma glutamyl transpeptidase; 7, Glucose; 8, Glutamic oxalacetic transaminase; 9, Glutamic pyruvic transaminase; 10, Inorganic phosphorus; 11, Lactate Dehydrogenase; 12, Magnesium; 13, Total protein; 14, Uric acid; 15, Blood urea nitrogen; 16, Total bilirubin; 17, Triglyceride; 18, Creatine Kinase; 19, Sodium; 20, Potassium; 21, Chloride

### Gold Distribution in Tissue

Increases in gold concentrations in the lung tissue from the high dose group as compared to controls were statistically significant (*p *< 0.01) and increased with dose in both the males and females (Table 8 and 9). Gold concentrations in kidneys also increased in a dose dependent manner with statistical significance (p < 0.01). Interestingly, there was gender-related difference in gold concentrations in the kidneys (Table 8 and 9), with female kidneys showing more gold accumulation than the male kidneys. Apart from the lungs and kidneys, no dose-dependent increase of gold nanoparticles was found in the blood, liver, or olfactory bulb from both the male and female rats. Gold levels in blood were less than the tissues measured so that any small amount of contamination of other tissues with blood or blood contained in tissue was unlikely to greatly influence tissue measurements of gold content. There was a significant increase in gold nanoparticle concentration only in brain tissue from high-dose females (Table 9).

**Table 8:** Gold distribution in tissue of male rats after 90-day gold nanoparticle exposure.

GROUP: (mean ± S.E)	Control	Low	Middle	High
Lungs (ng/g)	14.33 ± 1.25 (5)	17.41 ± 1.49 (5)	36.88 ± 1.69 (5)	2191.77 ± 222.59^a ^(5)
Liver (ng/g)	13.58 ± 0.69 (5)	13.72 ± 1.26 (5)	13.77 ± 1.62 (5)	14.34 ± 1.56 (5)
Kidneys (ng/g)	18.56 ± 1.37 (5)	21.19 ± 0.75 (5)	23.13 ± 1.33 (5)	43.24 ± 2.15^a ^(5)
Brain (ng/g)	17.62 ± 1.71 (5)	19.77 ± 0.96 (5)	15.03 ± 1.10 (5)	17.02 ± 0.94 (5)
Olfactory bulb (ng/g)	11.07 ± 3.88 (8)	19.22 ± 3.61 (10)	21.37 ± 3.65 (10)	16.57 ± 3.47 (8)
Blood (ng/ml)	6.93 ± 0.61 (8)	5.75 ± 0.73 (10)	6.41 ± 0.65 (10)	6.86 ± 0.89 (8)

( ): number of animals, a: p < 0.01, high group vs. other groups

**Table 9:** Gold distribution in tissue of female rats after 90-day gold nanoparticle exposure.

GROUP: (mean ± S.E)	Control	Low	Middle	High
Lungs (ng/g)	13.75 ± 0.95 (5)	15.07 ± 2.02 (5)	28.34 ± 1.16 (5)	1960.02 ± 92.80^a ^(5)
Liver (ng/g)	16.57 ± 3.89 (5)	12.08 ± 2.13 (5)	12.31 ± 1.18 (5)	14.96 ± 1.54 (5)
Kidneys (ng/g)	21.27 ± 0.65 (5)	19.43 ± 0.79 (5)	26.37 ± 0.79 (5)	61.90 ± 3.15^a,** ^(5)
Brain (ng/g)	19.13 ± 2.31 (5)	18.42 ± 1.07 (5)	17.87 ± 0.86 (5)	22.15 ± 0.75** (5)
Olfactory bulb (ng/g)	23.19 ± 2.45 (10)	20.22 ± 4.35 (10)	23.56 ± 4.15 (10)	22.92 ± 4.07 (10)
Blood (ng/ml)	6.66 ± 0.78 (10)	6.92 ± 1.08 (10)	5.10 ± 0.58 (10)	8.98 ± 1.25 (10)

( ), number of animals; a, p < 0.01 high group vs. other groups; **, p < 0.01 female rats vs. male rats in high group

### Pulmonary Inflammation

**Figure 6:**
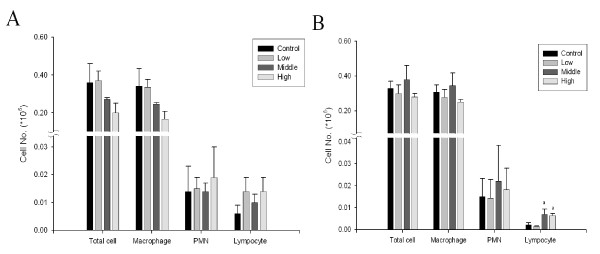
**Number of total cells, macrophages, PMN, and lymphocytes in rats exposed to gold nanoparticles**. A, male; B, female. (B. a, p < 0.05 middle and high groups vs. control and low groups)

Total number of cells, alveolar macrophages, polymorphonuclear cells (PMN), and lymphocytes in BAL fluid after 90-days of exposure are shown in Figure 6. When compared to the control group, there were no significant changes in total cell numbers, alveolar macrophages, PMN, or lymphocytes of any exposed males. There was a significant increase in lymphocytes in middle and high-dose females (Figure 6B). Albumin, LDH, and total protein in BAL did not increase significantly in either male or female rats (data not shown).

### Pulmonary Function Testing

**Figure 7:**
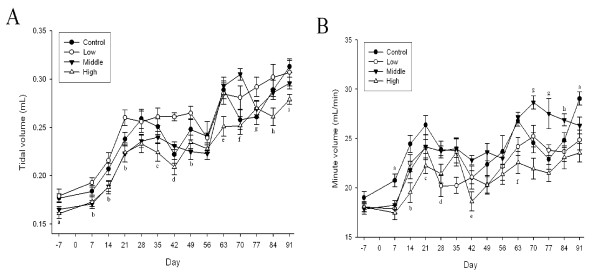
**Lung function test of male rats after gold nanoparticle exposure**. A: Tidal volume (mL), B: Minute volume (mL/min) (A. a, p < 0.05, high group vs. control and low groups; b, p < 0.05, middle and high groups vs. low group; c, p < 0.05, high group vs. control and middle groups; d, p < 0.01, high group vs. low and middle groups; e, p < 0.05, high group vs. other groups; f, p < 0.01, control and high groups vs. middle group; g, p < 0.05, control and high groups vs. low group; h, p < 0.05, high group vs. low group; i, p < 0.05, high group vs. control group. B. a, p < 0.01, control group vs. other groups; b, p < 0.01, high group vs. control and low groups; c, p < 0.05, low and high groups vs. control group; d, p < 0.05, low group vs. control and middle groups; e, p < 0.05, high group vs. middle group; f, p < 0.01, low and high groups vs. control and middle groups; g, p < 0.01, middle group vs. other groups; h, p < 0.05, middle group vs. low and high groups)

**Figure 8:**
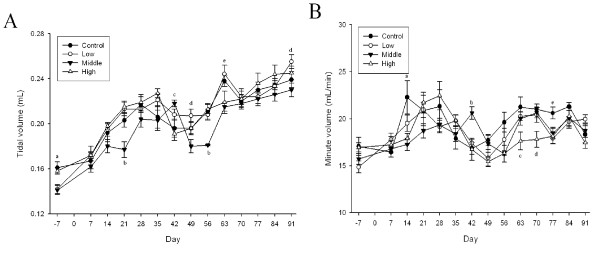
**Lung function test of female rats after gold nanoparticle exposure**. A, Tidal volume (mL); B, Minute volume (mL/min) (A. a, p < 0.01, control and high groups vs. low and middle groups; b, p < 0.01, middle group vs. other groups; c, p < 0.01, middle group vs. high group; d, p < 0.05, low group vs. middle group; e, p < 0.01, low group vs. middle and high groups. B. a, p < 0.01, control group vs. middle and high groups; b, p < 0.01, middle group vs. other groups; c, p < 0.05, high group vs. other groups; d, p < 0.01, high group vs. other groups; e, p < 0.05, control group vs. other group)

Among the pulmonary function test parameters, there were significant changes in tidal volume and minute volume during the 90-days of gold nanoparticle exposure (*p *< 0.01-0.05) (Figures 7,A and 7B and 8,A and 8B). Dose-dependent tidal volume decreases in male rats led to minute volume decreases in the high-dose animals. A tendency towards a dose-dependent decrease in the tidal volume appeared to be present in female rats, but was not statistically significant.

### Histopathologic Examination

**Figure 9:**
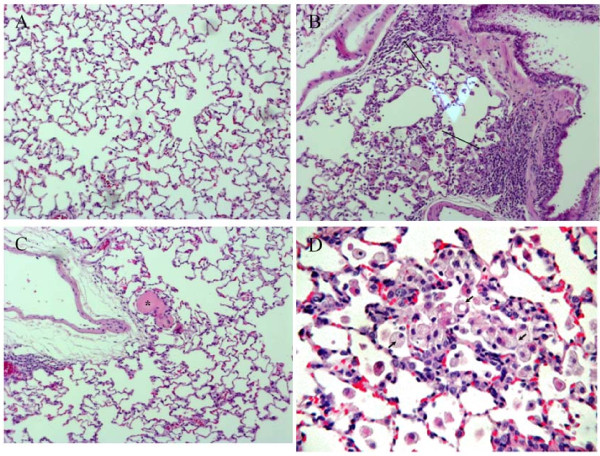
**Histopathologic findings for lungs**.

The only significant changes in histopathology occurred in lungs (Table 10 and 11), where there was minor focal inflammation with an inflammatory infiltrate of mixed cell type (lymphocyte/neutrophil/macrophage) were noted in both treated male and female rats. The increases were dose-dependent in female rats (Figure 9).

**Table 10:** Histopathologic observations for male rats.

GROUP:					Control		Low		Middle		High	
Number of Animals					8		10		10		8	
					N	%	N	%	N	%	N	%

	No microscopic findings				8/8	100	10/10	100	6/10	60	5/8	62.5
	
	Abnormality*				0/8	0	0/10	0	4/10	40	3/8	37.5
	
Liver		Inflammation	Focal	minimum	0/8	0	0/10	0	0/10	0	1/8	12.5
			
	Sign		Multifocal	minimum	0/8	0	0/10	0	3/10	30	0/8	0
		
		Necrosis	Focal	minimum	0/8	0	0/10	0	0/10	0	2/8	25
		
		Vacuolation	Hepatocellular	minimum	0/8	0	0/10	0	1/10	10	1/8	12.5

	No microscopic findings				7/8	87.5	10/10	100	10/10	100	5/8	62.5
	
Lungs	Abnormality*				1/8	12.5	0/10	0	0/10	0	3/8	37.5
	
	Sign	Inflammation*	Focal	minimum	1/8	12.5	0/10	0	0/10	0	3/8	37.5
		
		Osseous Metaplasia			0/8	0	0/10	0	0/10	0	1/8	12.5

*, p < 0.05 compared with controls. Abnormality" refers to such changes as inflammation, vacuolization and necrosis upon histopathological examination. One abnormality is counted even if inflammation and necrosis are present simultaneously.

**Table 11:** Histopathologic observations for female rats.

GROUP:					Control		Low		Middle		High	
Number of Animals					10		10		10		10	
					N	%	N	%	N	%	N	%

	No microscopic findings				9/10	90	9/10	90	8/10	80	7/10	70
	
	Abnormality				1/10	10	1/10	10	2/10	20	3/10	30
	
		Inflammation	Focal	minimum	0/10	0	1/10	10	0/10	0	0/10	0
				
Liver				mild	1/10	10	0/10	0	0/10	0	0/10	0
		
	Sign	Necrosis	Focal	minimum	0/10	0	0/10	0	1/10	10	1/10	10
		
		Vacuolization	Hepatocellular	minimum	0/10	0	0/10	0	1/10	10	2/10	20
				
				mild	0/10	0	0/10	0	0/10	0	1/10	10

	No microscopic findings				10/10	100	10/10	100	9/10	90	3/10	30
	
Lungs	Abnormality**				0/10	0	0/10	0	1/10	10	7/10	70
	
	Sign	Inflammation**	Focal	minimum	0/10	0	0/10	0	1/10	10	6/10	60
				
				mild	0/10	0	0/10	0	0/10	0	1/10	10

**, p < 0.01, compared with control. Abnormality" refers to such changes as inflammation, vacuolization and necrosis upon histopathological examination. One abnormality is counted even if inflammation and necrosis are present simultaneously

### Erythrocyte aggregation and kidney function test

**Figure 10:**
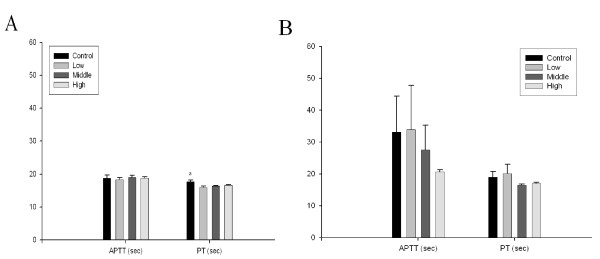
**Blood coagulation time (seconds) for rats exposed to gold nanoparticles**. A, male; B, female. (A. a, p < 0.05 control group vs. other groups)

**Figure 11:**
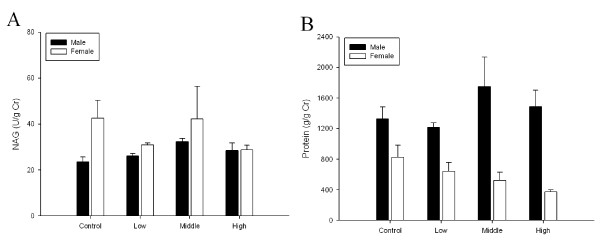
**Kidney function test after 90-day gold nanoparticle exposure**. A, NAG; B, Protein

To evaluate changes in red blood cell aggregation or blood coagulation, erythrocyte aggregation, activated partial thromboplastin time (APPT), and prothrombin time (PT) were tested. No significant differences were found between the control and any treated animals in APPT or PT (Figure 10,A and 10B). There were no significant differences among dose groups in kidney function as measured based on the NAG and protein in the urine (Figure 11,A and 11B).

## Discussion

To the best of our knowledge, this is the first subchronic 90-day inhalation study using gold nanoparticles to be reported in the peer-reviewed literature. An important aspect of this and predecessor studies from our laboratory is that the surface of the nanoparticles used for this exposure were not modified and not oxidized. There is one previous inhalation (intratracheal instillation) study which used 1.4 nm and 18 nm particles whose surface was ligated with Ph_2_PC_6_H_4_SO_3_Na [23] and one 15-day whole body inhalation study which used citrated 20 nm particles [24].

Interestingly, a recent publication [25] found that colloidal gold nanoparticles diluted in ultrapure water were well-dispersed, while agglomerates were formed when the diluent was phosphate buffered saline. In that study, rats were administered 50 nm and 250 nm gold particles by intratracheal injection. Despite differences in the degree of agglomeration due to the two diluents, no major differences in pulmonary and systemic toxicity markers were observed.

It is possible to calculate a deposited dose in the lung for this study. The deposited dose is calculated as follows:

If the minute volume of male and female Sprague-Dawley rats in a subchronic study is 0.19 and 0.15 L/min, respectively [26] and the fraction of the dose deposited for particles less than 10 nm in diameter is 0.8 [27], then the deposited dose to the lung would be 0.14 μg, 1.35 μg and 71.1 μg for the low, middle, and high dose males, respectively. For the low, middle, and high dose females, the deposited dose to the lung would be 0.11 μg, 1.07 μg and 56.2 μg, respectively. For comparison purposes and using the same assumptions, we can calculate deposited dose to the lung from our previously reported subchronic inhalation study of 18-19 nm silver particles [14]. The deposited dose to the lung for the silver study would be 170 μg, 470 μg and 1800 μg for the low, middle, and high dose males, respectively. For the low, middle, and high dose females, the deposited dose to the lung would be 140 μg, 370 μg and 1400 μg, respectively. Thus, the high deposited dose in our subchronic gold study is about 40% by weight of the low deposited dose in our subchronic silver study.

There is considerable uncertainty in the fraction of the dose retained for particles less than 10 nm in diameter as opposed to the fraction of the dose deposited as used in the calculation above. It is likely that the retained dose is significantly different than 0.8 due to such factors as translocation to other organs and species-specific deposition patterns. The reader is referred to more comprehensive discussions of retention of nanoparticles and deposition modeling in human and animal models [28-32].

Whole body inhalation studies involve an additional component of exposure; that of ingestion since particles accumulate on the skin and fur. It is well known that rats clean skin and fur by licking, thus introducing particles to the gastrointestinal tract. For particles of gold larger than the nanometer range, it is generally assumed that the particles are not absorbed. Little is known about gastro-intestinal absorption of gold nanoparticles, but there are reports on the permeability of rat intestine to colloidal gold nanoparticles. Sonavane et al. [33] studied movements of 15, 102, and 198 nm colloidal gold particles across intestine *in vitro*. Fifteen nm particles were shown to cross the intestine more readily than 102 or 198 nm particles. The permeation of these particles through rat intestine was higher than rat skin. Hillyer et al. [34] studied absorption of 4, 10, 28, and 58 colloidal gold nanoparticles fed to mice. In general, smaller particles were absorbed more readily and corresponding tissue levels were higher. The applicability of this data to whole body inhalation is not apparent because of the colloidal nature of the tested particles [33,34]. Clearly, quantitative *in vivo *data on the absorption of unmodified gold nanoparticles in the rat would be of great value in determining the relative contribution of gastrointestinal absorption to the accumulation of gold from nanoparticles.

Tissue concentrations of gold from control rats have been reported in several studies [24,35] and tend to be lower than found in our study (Table 8 and 9). Takenaka et al. [34] reported gold in control lungs to be 0.07 ± 0.003 ng/g and in control blood to be 0.1 ± 0.07 ng/ml (mean ± SD). Yu et al. [24] reported gold in control lungs to be 5.50 ± 4.44 ng/g and in control blood to be 5.83 ± 3.02 ng/ml. Yu et al. [24] also reported gold in control olfactory bulb to be 4.44 ± 4.43 ng/g and in control brain to be from 4.67 ± 3.63 to 20.27 ± 15.79 ng/g (presumed to be mean ± SD) depending on the part of the brain analyzed. Because of the low levels of gold in tissues, the authors cannot rule out the possibility of contamination of internal tissues from skin or fur. Mitigating that possibility is previous experience by the authors in whole-body inhalation studies involving nanoparticles. Nevertheless, the reader should consider the possibility for such contamination.

The results of this study indicated that the lungs were the major target tissue; pulmonary effects included a decrease in tidal and minute volume and the presence of mixed inflammatory cell infiltrates. Dose-related changes in tidal and minute volume tend to be obscured by changes over time which also occurred in control animals. In our experience, pulmonary function changes can reproduce poorly over time because they are dependent on so many variables. The decrease in pulmonary function following 90-days of gold nanoparticle inhalation in the current study was similar, although lower, than that reported after 90-days of silver nanoparticle inhalation [14].

In the present study inhaled gold nanoparticles accumulated in a dose-dependent manner in lungs and kidneys of both male and female rats (p < 0.01), but not in liver, blood, and olfactory bulb. This is in contrast to the study of Yu et al. [24] where rats are exposed to 20 nm gold nanoparticles at a concentration of 2 × 10^6^/cm^3 ^(mass concentration not reported) for 15 days by inhalation reported that the particles relocate from lungs to liver. Exposure in that study for 5 days resulted in a significant increase of gold in the lungs and olfactory bulb, as detected by ICP-MS; after 15 days of exposure, a significant accumulation of gold was detected in the lungs, esophagus, tongue, kidneys, aorta, spleen, septum, heart, and blood. Five or 15 days of inhalation exposure to gold nanoparticles resulted in a slight accumulation of gold in liver and a minimal increase the gold content of the olfactory bulb indicating a small but significant translocation from lung to blood after 15 days of exposure. Takenaka et al. [35] exposed rats to 16 nm gold particles at a concentration of 4 × 10^5^/cm^3 ^(88 μg/m^3^) for 6 hours and then serially sacrificed animals at 0, 1, 4, and 7 days. These data also indicate a small but significant translocation from lung to blood.

The lack of significant increase in gold concentration in the olfactory bulb in our study is interesting with regard to findings in previously reported studies of shorter duration, particularly those of Yu et al. [24]. They showed a small but significant increase in the concentration of gold in the olfactory bulb and parts of the brain. The reason for the differences between our study and that of Yu et al. [24] are not apparent, but could be related to the surface composition of the nanoparticle itself, duration of exposure or the higher concentrations of gold in found in the olfactory bulbs of control animals.

The results of our 90-day study are not consistent with data from studies of nanoparticles of different composition and duration and stand in contrast to Elder et al. [36] using inhaled Mn oxides nanoparticles and Balasubramanian et al. [37] using intravenously injected gold nanoparticles. Elder et al. found that when rats inhaled manganese oxide particles (agglomerates measured 30 nm in diameter with primary particles of 3-8 nm) for 12 days, the particles accumulated in the olfactory bulb [36]. It is not clear if the differences between our study and that of Elder et al. are due to exposure duration, particle size, or solubility. Balasubramanian et al. [37] reported that a single intravenous injection of gold nanoparticles yielded a large amount of gold in the olfactory bulb (up to 72.2 ng/g) two months after intravenous gold nanoparticle injection. A similar comparative distribution between intravenous injection and intratracheal instillation was also observed in a study by Semmler-Behnke et.al. [23]. Perhaps this concurrence of distribution is related to the fact that both intravenous injection and intratracheal installation deliver a large bolus of nanoparticles whereas normal inhalation delivers a much lower concentration of nanoparticles per unit time.

Throughout this study, male rats were larger than females (Tables 2 and 3, Figures 5A and 5B). There appeared to be no consistent differences between male and female in the organ content of gold and particularly lung indicating that differences in were not likely to have been due to a gender difference in deposition.

The higher accumulation of gold nanoparticles in the kidneys found in the present study was also previously observed in silver nanoparticle inhalation and oral exposure studies [14,16], suggesting that the kidneys are the major accumulation site for metal nanoparticles whether the particles are ionized, like silver, or non-ionized, like gold. Gender-related accumulation of gold nanoparticles noted in this study was also observed for silver nanoparticles in the inhalation and oral exposure studies [14,16]. It therefore appears that at least over the range of average nanoparticle sizes of 5 nm (gold, inhalation), 15-20 nm (silver, inhalation), and up to 60 nm (silver, oral) there is a similar gender-related accumulation, and that there is a difference in the pattern of nanoparticle distribution between male and female kidneys. It is not apparent whether this difference is anatomically or hormonally-based.

In contrast to the 0-hypothesis for silver suggested by Wijnhoven et al. [38], in which silver toxicity mainly originates from silver ions generated from the surface of silver nanoparticles, the present results using gold nanoparticle inhalation provide a different view of nanoparticle toxicity and distribution. Gold nanoparticles (1.4 nm) injected intravenously or administered by intratracheal instillation are excreted into the urine and not likely ionized in the body, they are translocated to tissues as particles rather than in an ionic form [23]. This implies that part or even a majority of the tissue distribution pattern of silver nanoparticles could be due to translocation in particulate form rather than ionic form and that most of the silver captured in kidneys is not in ionic form.

Currently, there are no occupational exposure standards for gold dust, fumes, or nanoparticles other than general particulate standards such as the "particles not otherwise regulated" standard of the US OSHA permissible exposure limit (PEL) of 5 mg/m^3^ for respirable particles. Changes observed in lung histopathology and function in high-dose animals appear in and of themselves to be minor. In the previously reported study on silver nanoparticles, similar changes were noted [14] and the authors interpreted them to be transient and not sufficient to establish an effects level. It appears that our original interpretation was not correct since when rats from a recent 12 week exposure study similar to Sung et al. [15] were allowed to "recover," decreases in tidal and minute volumes from the middle and high dose groups have persisted up to 12 weeks post-exposure (unpublished data). In light of similar changes in this study, it appears that the highest concentration (20 μg/m^3^) is a LOAEL and the middle concentration (0.38 μg/m^3^) as a NOAEL.

## Competing interests

The authors declare that they have no competing interests.

## Authors' contributions

IJY headed the study and performed the pathology together with MYS, YHC, BSH, JHJ and HKC performed the pathology peer-review. JHJ, JUY, and KSJ generated and monitored gold nanoparticles in the inhalation chamber. IJY drafted the manuscript with JHS and BK. JHS headed all animal treatments with MYS, KSS and HRR. JDP, DWK and JHL contributed tissues distribution study. IJY and BK conceived and designed the study. All authors reviewed and interpreted data and read and approved the final manuscript
